# EGFR activation triggers cellular hypertrophy and lysosomal disease in NAGLU-depleted cardiomyoblasts, mimicking the hallmarks of mucopolysaccharidosis IIIB

**DOI:** 10.1038/s41419-017-0187-0

**Published:** 2018-01-18

**Authors:** Valeria De Pasquale, Antonio Pezone, Patrizia Sarogni, Alfonso Tramontano, Gabriele Giacomo Schiattarella, Vittorio Enrico Avvedimento, Simona Paladino, Luigi Michele Pavone

**Affiliations:** 1 Department of Molecular Medicine and Medical Biotechnology, University of Naples Federico II, Via S. Pansini 5, Naples, 80131 Italy; 2 Department of Advanced Biomedical Sciences, University of Naples Federico II, Via S. Pansini 5, Naples, 80131 Italy

## Abstract

Mucopolysaccharidosis (MPS) IIIB is an inherited lysosomal storage disease caused by the deficiency of the enzyme α-*N*-acetylglucosaminidase (NAGLU) required for heparan sulfate (HS) degradation. The defective lysosomal clearance of undigested HS results in dysfunction of multiple tissues and organs. We recently demonstrated that the murine model of MPS IIIB develops cardiac disease, valvular abnormalities, and ultimately heart failure. To address the molecular mechanisms governing cardiac dysfunctions in MPS IIIB, we generated a model of the disease by silencing NAGLU gene expression in H9C2 rat cardiomyoblasts. NAGLU-depleted H9C2 exhibited accumulation of abnormal lysosomes and a hypertrophic phenotype. Furthermore, we found the specific activation of the epidermal growth factor receptor (EGFR), and increased phosphorylation levels of extracellular signal-regulated kinases (ERKs) in NAGLU-depleted H9C2. The inhibition of either EGFR or ERKs, using the selective inhibitors AG1478 and PD98059, resulted in the reduction of both lysosomal aberration and hypertrophy in NAGLU-depleted H9C2. We also found increased phosphorylation of c-Src and a reduction of the hypertrophic response in NAGLU-depleted H9C2 transfected with a dominant-negative c-Src. However, c-Src phosphorylation remained unaffected by AG1478 treatment, posing c-Src upstream EGFR activation. Finally, heparin-binding EGF-like growth factor (HB-EGF) protein was found overexpressed in our MPS IIIB cellular model, and its silencing reduced the hypertrophic response. These results indicate that both c-Src and HB-EGF contribute to the hypertrophic phenotype of NAGLU-depleted cardiomyoblasts by synergistically activating EGFR and subsequent signaling, thus suggesting that EGFR pathway inhibition could represent an effective therapeutic approach for MPS IIIB cardiac disease.

## Introduction

Mucopolysaccharidoses (MPSs) are a group of inherited metabolic diseases, belonging to the class of lysosomal storage diseases, caused by the deficiency of lysosomal enzymes required to metabolize glycosaminoglycans (GAGs)^1^. The GAG family includes four main subgroups: hyaluronic acid or hyaluronan (HA), keratan sulfate (KS), chondroitin/dermatan sulfate (CS/DS), and heparan sulfate (HS)/heparin. With the exception of HA, GAGs are covalently attached to a core protein, forming the so-called proteoglycans that are abundantly expressed on the cell surface and extracellular matrix^2^. Deficits of GAG degrading enzymes are responsible for seven different MPSs (MPS I, II, III, IV, VI, VII, and IX), where the accumulation of the undegraded GAG results in multiple organ dysfunctions, with distinct clinical manifestations depending on the type of the lacking enzyme and the accumulated substrate^1^. MPS patients show profound mental retardation, intractable behavior, skeletal abnormalities, cardiovascular dysfunction, and death in the second decade of life^1^. Cardiac disease has been commonly observed in MPS patients and contributes to the early mortality of these patients^3–6^.

Among MPS subtypes, the MPS IIIB is an autosomal recessive disorder caused by the deficiency of the α-*N*-acetylglucosaminidase (NAGLU) enzyme involved in HS degradation. The availability of MPS IIIB animal models and cultured cell systems, including primary fibroblasts from MPS IIIB patients^7,8^, has allowed to identify the defective lysosomal clearance of undigested HS as the primary cause of organ dysfunctions associated with the disease. Indeed, the defective lysosomal clearance of HS leads to the accumulation of abnormal levels of HS not only within the lysosomes of the cells, but also on the cell surface and in the extracellular matrix^8–10^, accounting for a number of pathologic events affecting various tissues and organs, including brain, liver, spleen, kidney, and heart^11–14^. Both during development and adult life, HS-associated proteoglycans (HSPGs) play crucial roles in the regulation of many physiological processes due to their property to bind and interact with signaling proteins, morphogens, growth factors, and other critical molecules^2,14–17^. Besides, the excess of extracellular HS has been shown to impair fibroblast growth factor 2 (FGF-2) receptor binding and signaling in cells derived from MPS I patients^10^, and to bind and sequester CXCL12-limiting hematopoietic migration in the murine model of MPS I^9^. As to date no treatments are available for MPS IIIB, a better understanding of the molecular mechanisms underlying HS storage defects at cellular level is a mandatory need to identify new targets for effective therapeutic approaches for MPS IIIB.

Although progressive and severe neurological disorders are the most relevant clinical manifestations of the MPS IIIB, cardiac defects including heart failure, arrhythmias, pulmonary hypertension, and coronary occlusion have also been reported in affected patients^3,18–23^. Indeed, we recently demonstrated that the murine model of MPS IIIB^7^ develops heart disease, valvular abnormalities, and heart failure^24^. However, the mechanisms governing cardiac dysfunction in MPS IIIB are still poorly investigated. In the present work, we generated a cellular model of MPS IIIB cardiac disease by silencing NAGLU gene expression in H9C2 rat cardiomyoblasts. This cell line has been proved to represent a relevant model to investigate mechanisms and consequences of cardiac diseases, including hypertrophy, and cardiomyocytes differentiation processes^25,26^. NAGLU-silenced H9C2 stable clones showed lysosomal abnormalities and hypertrophic phenotype, which correlated with epidermal growth factor receptor (EGFR) activation. Thus, we investigated the EGFR signaling pathway promoting cell pathology and hypertrophy in NAGLU-silenced cardiomyoblasts, providing new insights into the molecular mechanisms by which NAGLU deficiency and subsequent HS accumulation cause cardiac defects in MPS IIIB disease.

## Results

### NAGLU silencing induces lysosomal defects in H9C2 cardiomyoblasts

**Fig. 1:**
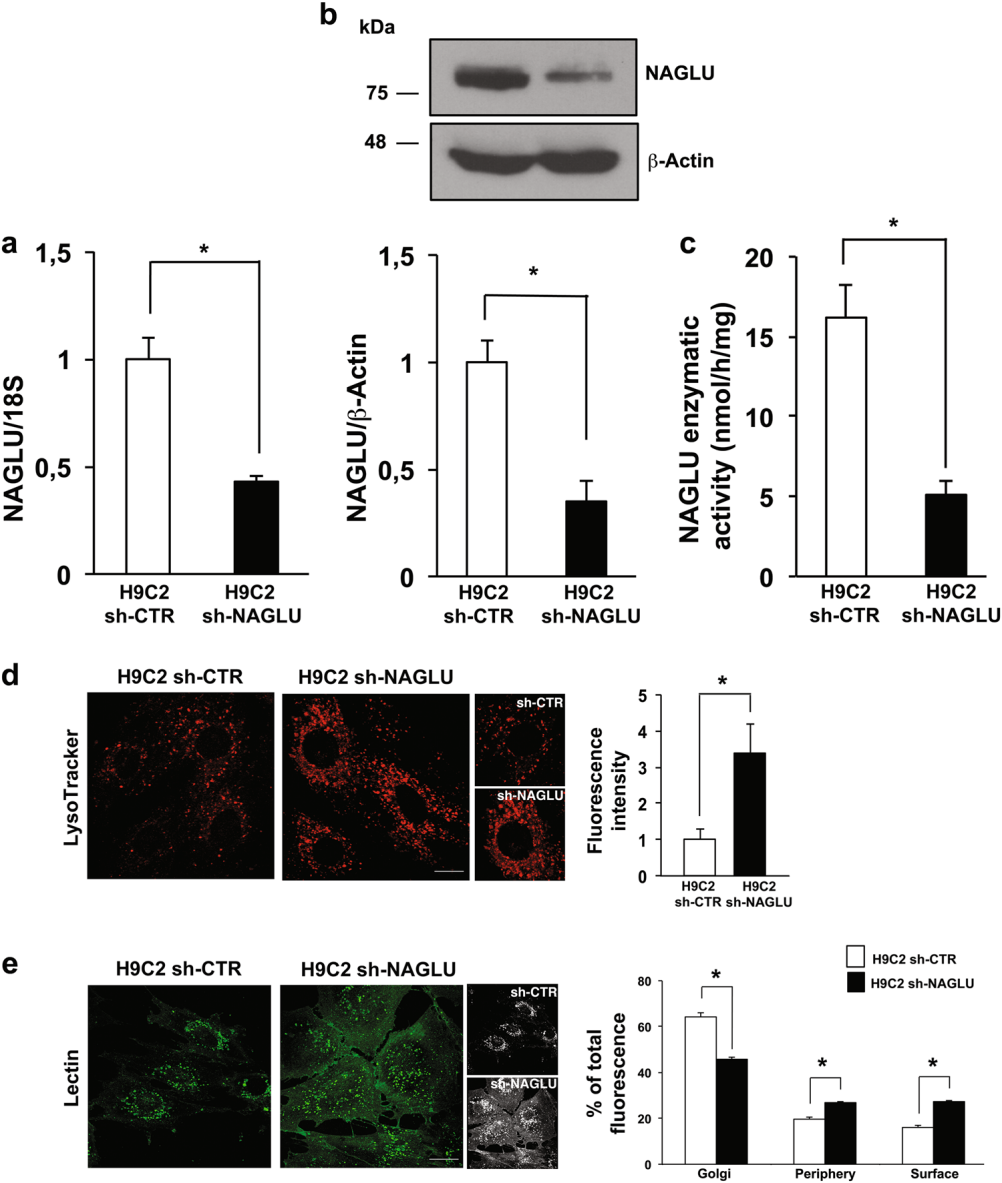
NAGLU silencing in H9C2 cardiomyoblasts causes lysosomal storage defects. **a** NAGLU mRNA expression levels in control H9C2 stably transfected with a non-targeting shRNA (H9C2 sh-CTR) and selected NAGLU-depleted H9C2 stable clones (H9C2 sh-NAGLU) as measured by quantitative RT-PCR analysis. The amount of NAGLU mRNA was normalized with respect to the amount of 18S ribosomal RNA housekeeping gene. The data reported are the mean ± SD of three independent experiments. *P < 0.05. **b** NAGLU protein levels in H9C2 sh-CTR and H9C2 sh-NAGLU as measured by western blotting analysis. To monitor equal loading of protein in the gel lanes, the blot was probed using anti-β-actin antibody. The data reported are the mean ± SD of three independent experiments of equal design. Densitometric analysis of the bands was performed, and the data obtained are reported on the histogram below. *P < 0.05. **c** NAGLU enzymatic activity in the extracts from H9C2 sh-CTR and H9C2 sh-NAGLU clones. Cell lysates were assayed for protein content and enzyme activity (mean ± SD, *n* = 3) as described in “Materials and methods” section. *P < 0.05. **d** Representative images of lysosomes labeled with LysoTracker probe in H9C2 sh-CTR and H9C2 sh-NAGLU. Higher magnification pictures are shown (small panels). Scale bar: 6 μm. In NAGLU-depleted H9C2 more abundant and larger intracellular vacuoles than control H9C2 are visible. Quantification of LysoTracker staining is the mean ± SD of three independent experiments. *P < 0.05. **e** Representative images of HS labeled by lectin-FITC in H9C2 sh-CTR and H9C2 sh-NAGLU. 3D reconstructions of confocal sections along *Z*-axis are shown (black and white panels). Scale bar: 10 μm. In NAGLU-depleted H9C2, an increased accumulation of HS in the periphery of the cells and on the cell membrane is detected compared to control H9C2. Quantification of HS staining is the mean ± SD of three independent experiments. *P < 0.05

To explore the molecular mechanisms underlying cardiac disease in MPS IIIB, we generated a cellular model of the disease by silencing NAGLU gene expression in H9C2 cardiomyoblasts through a pool of three DNA plasmids carrying different shRNAs against NAGLU mRNA. Silenced clones (H9C2 sh-NAGLU) showed a significant reduction of NAGLU mRNA and protein levels compared to that of H9C2 stably transfected with a non-targeting shRNA (H9C2 sh-CTR) (Fig. 1a, b). The reduction of NAGLU mRNA and protein levels resulted in about 70% decrease of NAGLU catalytic activity in the H9C2 sh-NAGLU compared to that of H9C2 sh-CTR (Fig. 1c). Since impaired digestion of HS in MPS IIIB results in the accumulation of the undegraded substrate and consequent lysosomal enlargement, we investigated whether NAGLU depletion is sufficient to induce aberrant lysosomal accumulation in our cellular model. H9C2 sh-NAGLU showed more abundant and larger intracellular vacuoles compared to H9C2 sh-CTR as detected by LysoTracker probe staining (Fig. 1d). Consistently, a significant increase of lysosomal-associated membrane protein LAMP2^27^ levels were detected in H9C2 sh-NAGLU compared to H9C2 sh-CTR (Supplementary Figure S1a). Moreover, by using a fluorescent lectin that specifically detects sugars and glycoproteins containing β(1 → 4)-*N*-acetyl-d-glucosamine and indeed recognizes HS, we evaluated the distribution of HS in NAGLU-depleted H9C2. Interestingly, while in H9C2 sh-CTR the lectin signal was mainly observed in the Golgi apparatus, in the H9C2 sh-NAGLU clones it was prevalently localized in intracellular spots at cell periphery and on the cell surface, indicating a strong accumulation of HS in these compartments (Fig. 1e).

Collectively, these data demonstrate that H9C2 sh-NAGLU cardiomyoblasts exhibit the storage abnormalities observed in MPS IIIB disease, thereby identifying this model as a valuable tool to investigate the pathophysiology of MPS IIIB-related cardiac disease.

### NAGLU silencing induces hypertrophy in H9C2 cardiomyoblasts

**Fig. 2:**
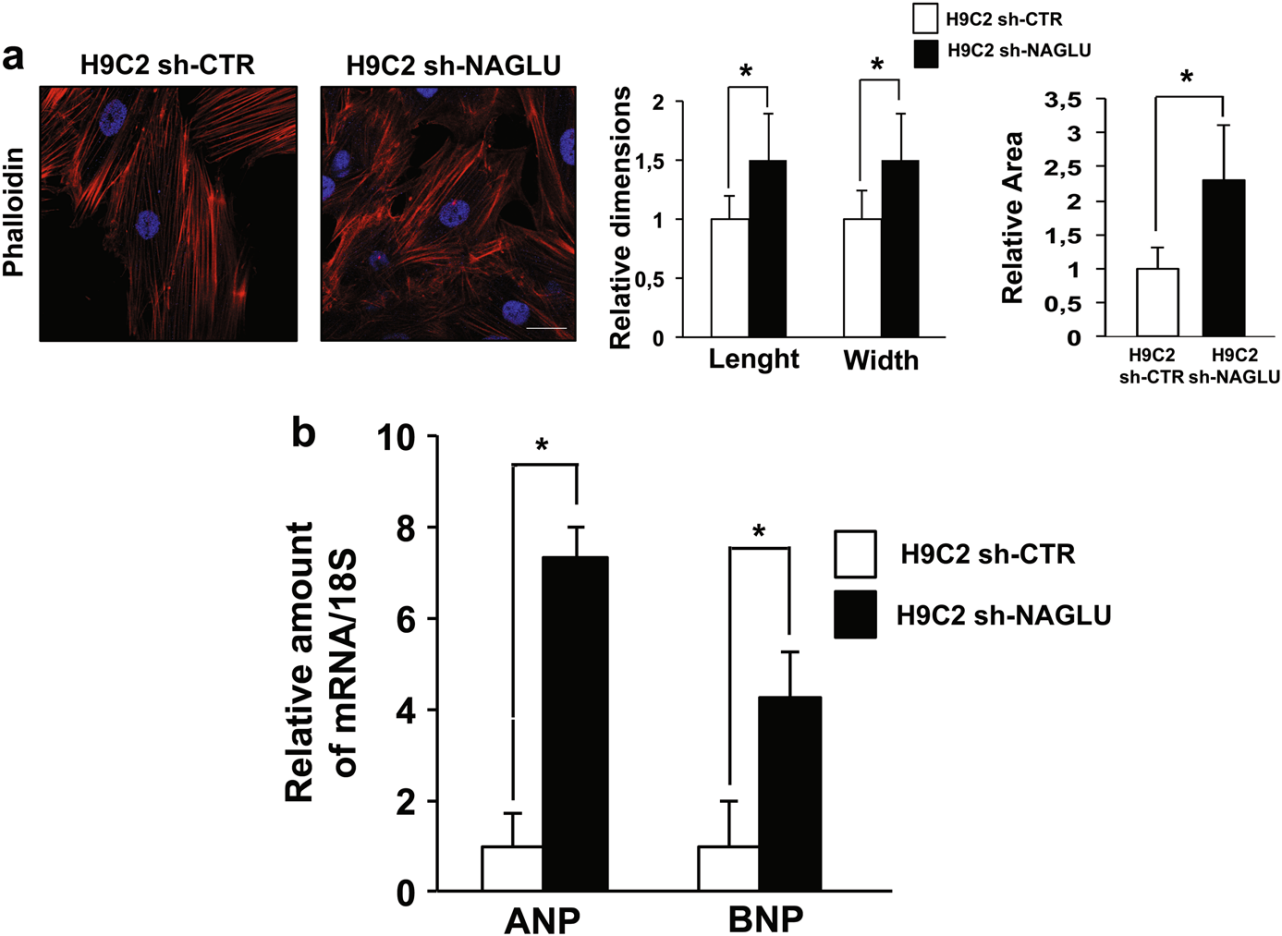
NAGLU silencing in H9C2 cardiomyoblasts promotes cellular hypertrophy. **a** Phalloidin staining of H9C2 sh-CTR and H9C2 sh-NAGLU actin cytoskeleton. Nuclei were counterstained with DAPI. Scale bar: 10 μm. Cell size enlargement was observed in NAGLU-depleted H9C2 compared to control H9C2. Quantification of cell size is the mean ± SD of three independent experiments. *P < 0.05. **b** Expression levels of mRNAs coding for ANP and BNP hypertrophic mediators in H9C2 sh-CTR and H9C2 sh-NAGLU as measured by quantitative RT-PCR analysis. The amount of ANP and BNP mRNA was normalized with respect to the amount of 18S ribosomal RNA housekeeping gene. The data reported are the mean ± SD of three independent experiments. *P < 0.05

We explored whether the lack of NAGLU might cause cellular hypertrophy as found in NAGLU^−/−^ mice^24^. To this aim, we labeled with fluorescent phalloidin the actin cytoskeleton of NAGLU-depleted and control H9C2 clones. Compared to H9C2 sh-CTR, the H9C2 sh-NAGLU showed a morphological expansion, typical of the hypertrophic phenotype, as evaluated by measuring length, width, and cell area (Fig. 2a). Accordingly, cell morphology was evaluated also by staining the clones with Coomassie blue, and H9C2 sh-NAGLU resulted larger in comparison to control clones (Supplementary Figure S1b). The hypertrophic phenotype of H9C2 sh-NAGLU was further confirmed by analyzing the expression of two markers of hypertrophy: atrial natriuretic peptide (ANP) and brain natriuretic peptide (BNP)^28^. ANP and BNP mRNA levels were significantly increased in H9C2 sh-NAGLU compared to control clones (Fig. 2b). Moreover, cell proliferation was impaired in NAGLU-depleted H9C2 (Supplementary Figure S1c, d) as well as the differentiation of H9C2 sh-NAGLU cardiomyoblasts to cardiomyocytes assessed by measuring the mRNA levels of the ventricular myosin light chain 2 (MLC2V) (Supplementary Figure S1e).

These data indicate that NAGLU deficiency leads to cellular hypertrophy, together with differentiation and growth defects.

### EGFR activation sustains hypertrophy and lysosomal defects in NAGLU-depleted H9C2 cardiomyoblasts

**Fig. 3:**
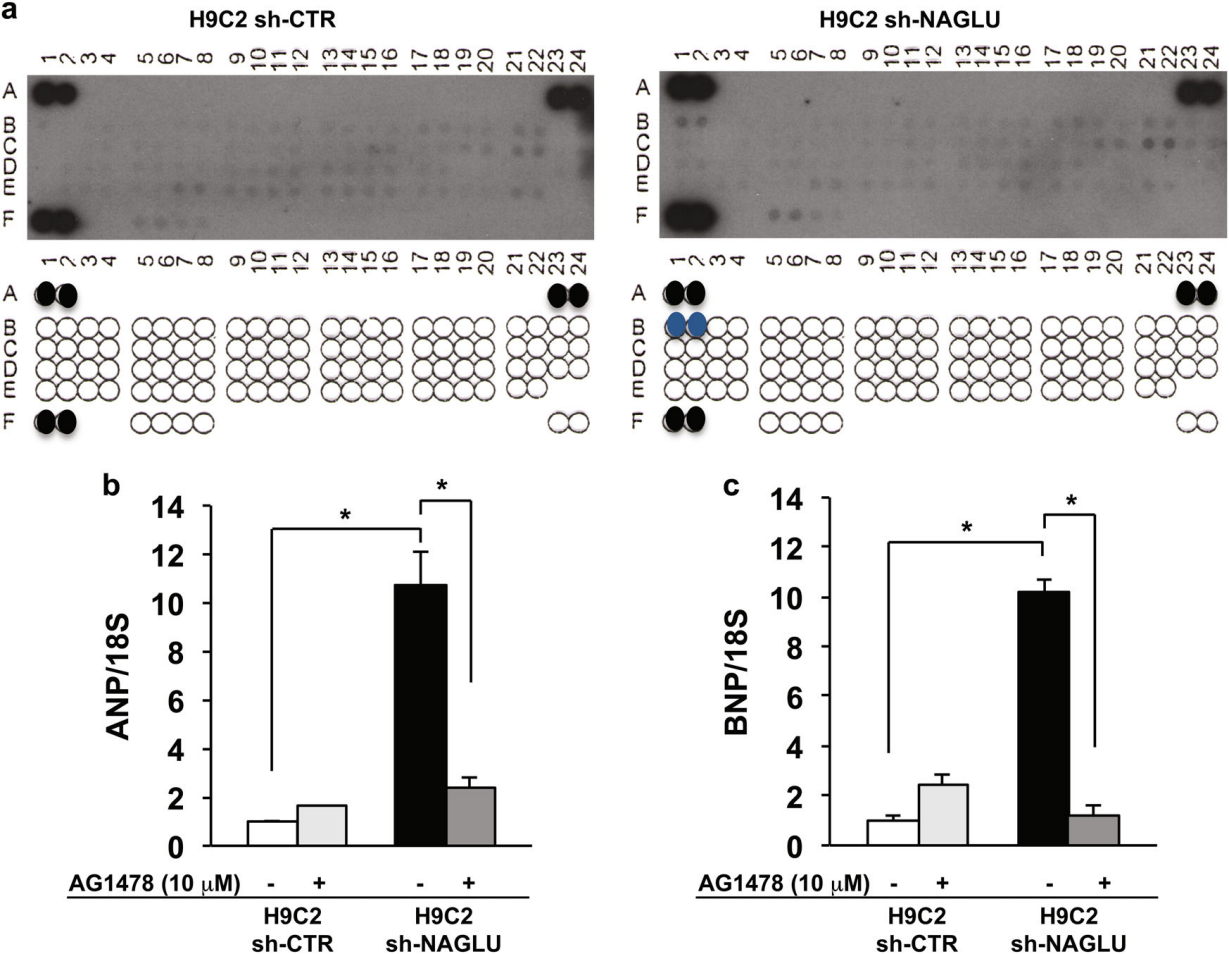
EGFR activation triggers hypertrophy in NAGLU-silenced H9C2 cardiomyoblasts. **a** Effect of NAGLU knockdown on RTK activity. p-RTK array showing the effect of NAGLU silencing on RTK phosphorylation. Cell lysates of H9C2 sh-CTR (left) and H9C2 sh-NAGLU (right) were applied to p-RTK arrays. The chemiluminescent film images (upper panels) are shown. The blue dots in the lower panels indicate the position of EGFR. **b**, **c** Expression levels of mRNAs coding for ANP (**b**) and BNP (**c**) in H9C2 sh-CTR and H9C2 sh-NAGLU both untreated and treated for 90 min with 10 μM of the EGFR inhibitor AG1478 as measured by quantitative RT-PCR analysis. The amount of ANP and BNP mRNA was normalized with respect to the amount of 18S ribosomal RNA housekeeping gene. The data reported are the mean ± SD of three independent experiments. *P < 0.05

**Fig. 4:**
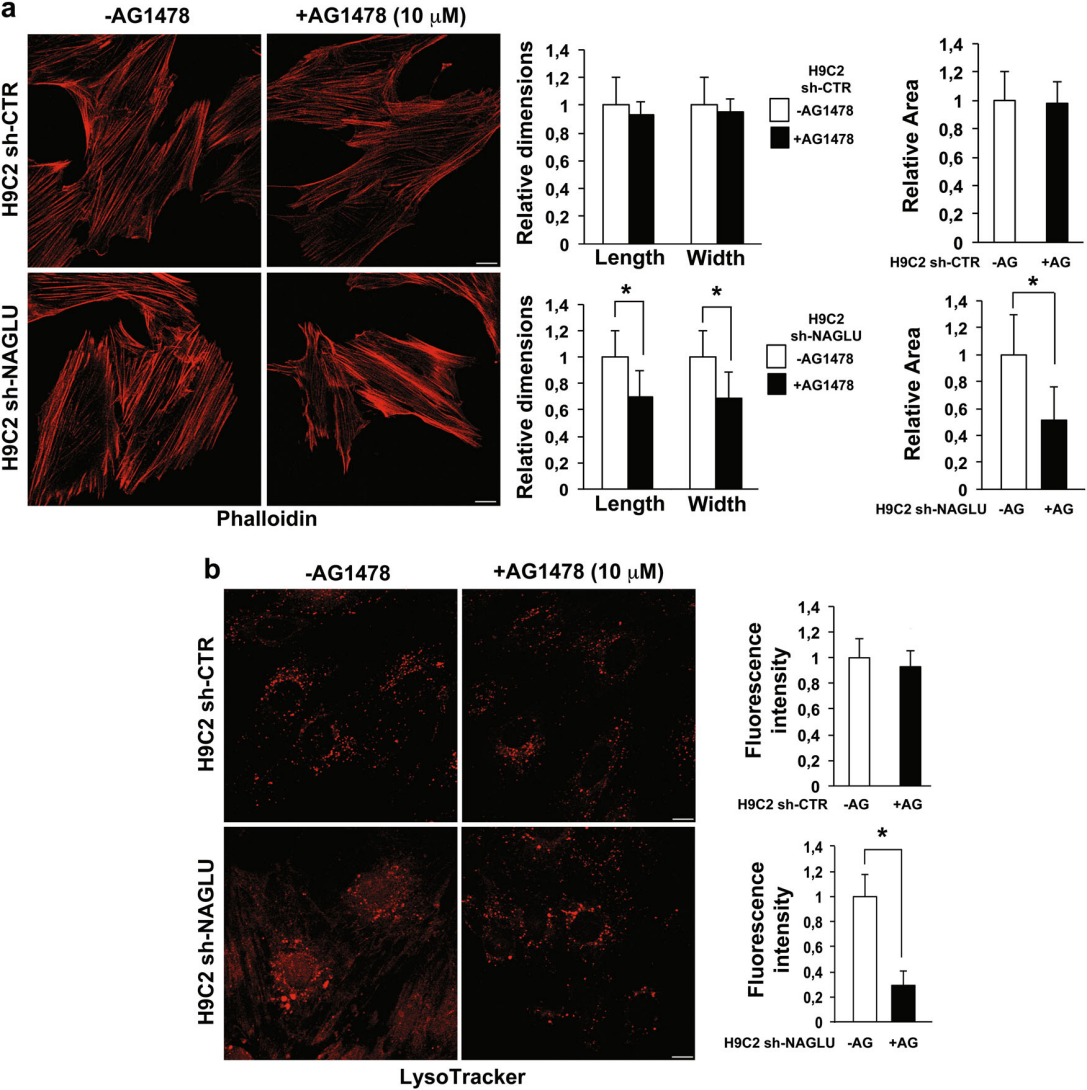
EGFR inhibition reduces the hypertrophic phenotype and lysosomal defects in NAGLU-silenced H9C2 cardiomyoblasts. **a** Representative images of actin cytoskeleton stained with phalloidin of H9C2 sh-CTR and H9C2 sh-NAGLU, both untreated and treated for 24 h with 10 μM of AG1478. Scale bars: 10 μm. A significant reduction of cell size was observed in AG1478-treated NAGLU-depleted H9C2 (lower panels), while no effect was detected in H9C2 sh-CTR clones (upper panels). Quantification of cell size is the mean ± SD of three independent experiments. *P < 0.05. **b** Representative images of the lysosomes labeled by LysoTracker probe in H9C2 sh-CTR and H9C2 sh-NAGLU, both untreated and treated for 24 h with 10 μM of AG1478. Scale bars: 10 μm. In the AG1478-treated H9C2 sh-NAGLU, lysosome staining was significantly reduced compared to the untreated clones (lower panels), while no effect was detected in H9C2 sh-CTR clones (upper panels). Quantification of LysoTracker staining is the mean ± SD of three independent experiments. *P < 0.05

In order to investigate the signaling pathways activated by NAGLU silencing in H9C2 cardiomyoblasts, we performed a phospho-receptor tyrosine kinase (RTK) array on both NAGLU-depleted and control H9C2 clones. Of 49 tested RTKs (Supplementary Table S1), the specific phosphorylation of EGFR in the H9C2 sh-NAGLU clones was detected as compared to control clones (Fig. 3a). This result was confirmed by western blotting analysis of the phosphorylation levels of EGFR in NAGLU-depleted H9C2 that resulted to be higher than control clones (Supplementary Figure S2a). Then, we evaluated EGFR involvement in the onset of the hypertrophic phenotype and lysosomal pathology in our cellular model. H9C2 sh-NAGLU clones treated with the specific EGFR inhibitor AG1478^29^ showed a significant reduction of ANP and BNP mRNA levels, as compared to the untreated clones (Fig. 3b, c). Moreover, AG1478 treatment did not affect cell size of H9C2 sh-CTR clones (Fig. 4a, upper panels) as measured by phalloidin staining, whereas it caused a significant reduction of cell dimensions of NAGLU-depleted H9C2 (Fig. 4a, lower panels). These results indicate that EGFR inhibition is able to rescue the hypertrophic phenotype in our MPS IIIB cellular model.

Besides, EGFR was already known to sustain GAG synthesis and to increase lysosomal pathology in different MPSs^30–32^. To establish the EGFR contribution to MPS IIIB lysosomal disorder in our cellular model, we evaluated the lysosomal accumulation in the H9C2 sh-CTR and H9C2 sh-NAGLU clones treated with the specific EGFR inhibitor AG1478. AG1478 treatment did not affect lysosomal compartments in H9C2 sh-CTR clones (Fig. 4b, upper panels), whereas it caused a significant reduction of the lysosomal staining in H9C2 sh-NALGU clones (Fig. 4b, lower panels) as measured by LysoTracker probe. Consistently, we measured LAMP2 protein levels in H9C2 sh-NAGLU and control clones in the presence of AG1478. Treatment with the EGFR inhibitor significantly reduced LAMP2 expression levels in NAGLU-depleted cardiomyoblasts, whereas it did not influence LAMP2 levels in control clones (Supplementary Figure S2b).

These findings demonstrate that the specific EGFR inhibition with AG1478 reduces both hypertrophy and lysosomal defects in our MPS IIIB cell model, providing more evidence for the involvement of EGFR activation in the development of cell pathology in MPS IIIB disease.

### EGFR activation promotes ERK1/2 phosphorylation in NAGLU-depleted H9C2 cardiomyoblasts

**Fig. 5:**
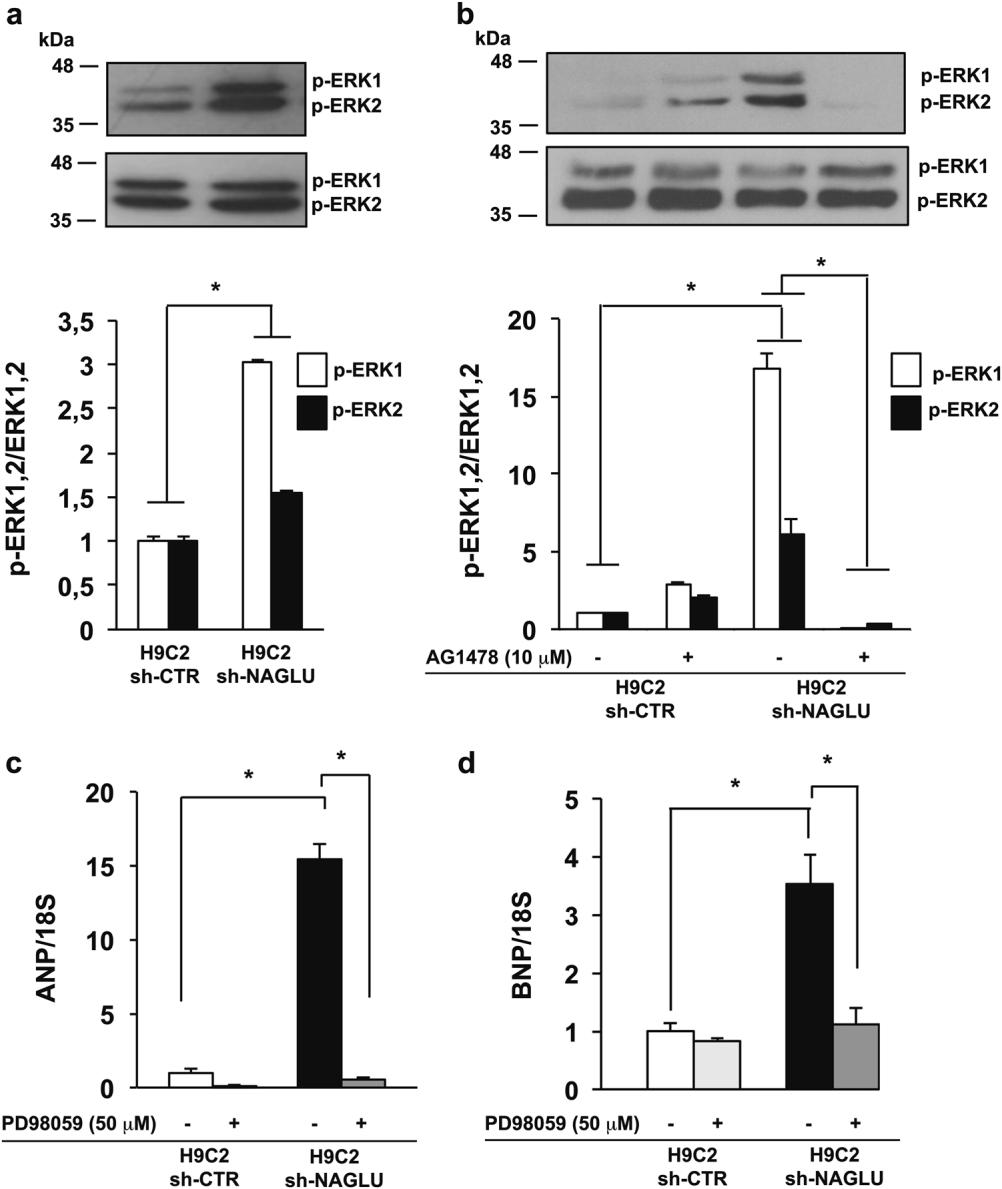
NAGLU silencing promotes ERK1/2 phosphorylation in H9C2 cardiomyoblasts. **a** ERK1/2 phosphorylation levels in H9C2 sh-CTR and H9C2 sh-NAGLU as measured by western blotting analysis. To monitor equal loading of protein in the gel lanes, the upper blot was stripped and reprobed using anti-ERK1/2 antibody. The data reported are the mean ± SD of three independent experiments of equal design. Densitometric analysis of the bands was performed, and the data obtained are reported on the histogram below. *P < 0.05. **b** EGFR inhibition reduces ERK1/2 phosphorylation in NAGLU-silenced H9C2. ERK1/2 phosphorylation levels in H9C2 sh-CTR and H9C2 sh-NAGLU both untreated and treated for 90 min with 10 μM of the EGFR inhibitor AG1478 as measured by western blotting analysis. The upper blot was stripped and reprobed using anti-ERK1/2 antibody. The data reported are the mean ± SD of three independent experiments of equal design. Densitometric analysis of the bands was performed, and the data obtained are reported on the histogram below. *P < 0.05. **c**, **d** Expression levels of mRNAs coding for ANP (**c**) and BNP (**d**) in H9C2 sh-CTR and H9C2 sh-NAGLU, both untreated and treated for 90 min with 50 μM of the MEK/ERK pathway inhibitor PD98059, as measured by quantitative RT-PCR analysis. The amount of ANP and BNP mRNA was normalized with respect to the amount of 18S ribosomal RNA housekeeping gene. The data reported are the mean ± SD of three independent experiments. *P < 0.05

**Fig. 6:**
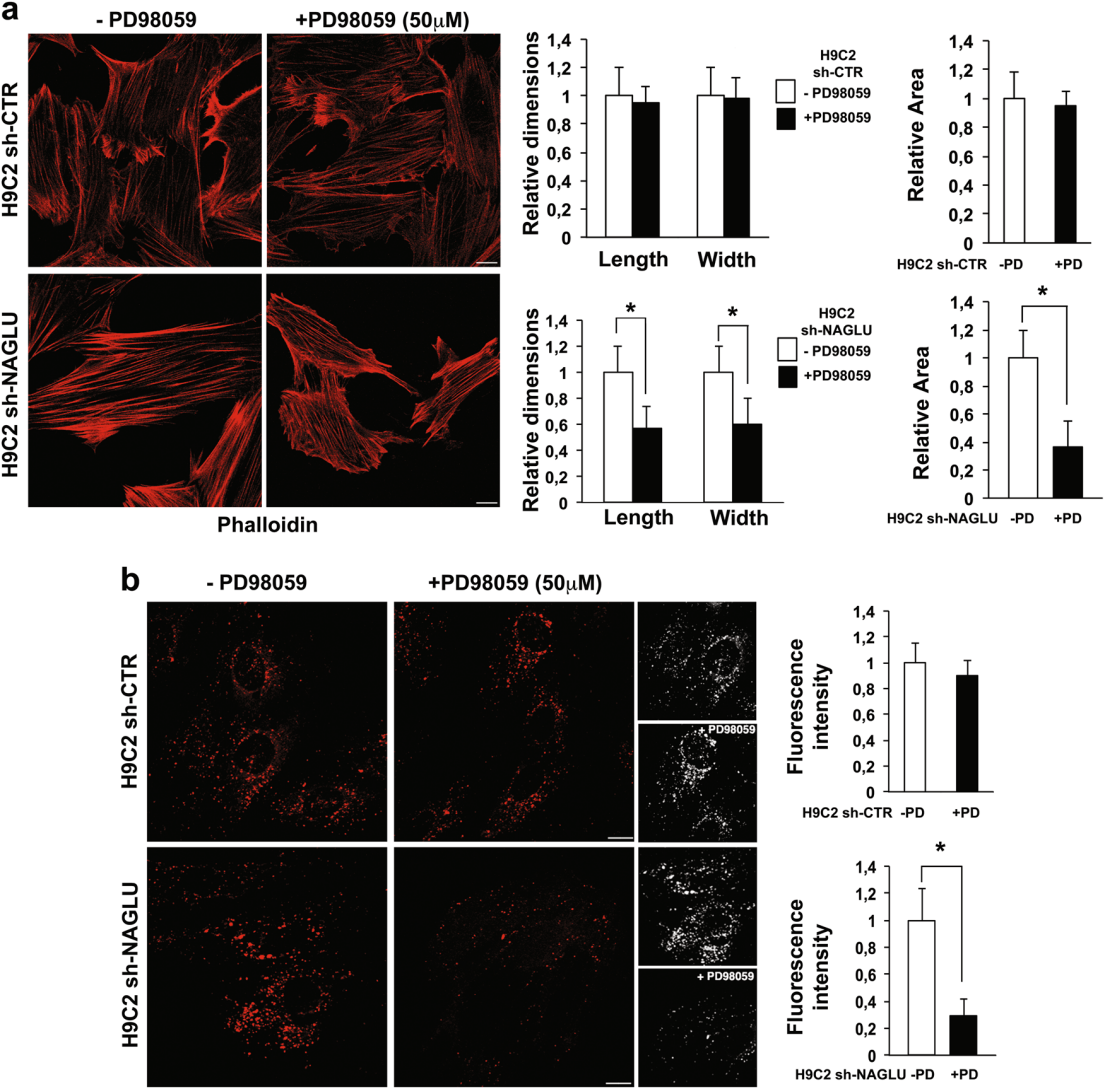
MEK/ERK inhibition reduces cell size enlargement and lysosome abnormalities in H9C2 sh-NAGLU. **a** Representative images of actin cytoskeleton stained with phalloidin of H9C2 sh-CTR and H9C2 sh-NAGLU clones, both untreated and treated for 24 h with 50 μM of PD98059. Scale bars: 10 μm. A significant reduction of cell size was observed in PD98059-treated NAGLU-depleted H9C2 (lower panels), while no effect was detected in H9C2 sh-CTR clones (upper panels). Quantification of cell size is the mean ± SD of three independent experiments. *P < 0.05. **b** Representative images of lysosomes stained with LysoTracker probe in H9C2 sh-CTR and H9C2 sh-NAGLU clones, both untreated and treated for 24 h with 50 μM of PD98059. 3D reconstructions of confocal sections along *Z*-axis are shown (black and white panels). Scale bars: 10 μm. In the PD98059-treated H9C2 sh-NAGLU clones, the fluorescence intensity was significantly reduced compared to the untreated clones (lower panels), while no effect was detected in H9C2 sh-CTR clones (upper panels). Quantification of LysoTracker staining is the mean ± SD of three independent experiments. *P < 0.05

The demonstration of the important role of EGFR in the induction of a hypertrophic response in NAGLU-depleted cardiomyoblasts prompted us to explore the EGFR signal transduction pathway contributing to the cardiac phenotype in our MPS IIIB cellular model. Among the downstream pathways activated by EGFR, phosphorylation of extracellular signal-regulated kinases (ERK1/2) has been considered the essential regulator of the hypertrophic response^33^. Therefore, we investigated the phosphorylation levels of ERK1 and ERK2 in NAGLU-depleted rat cardiomyoblasts. Western blot analysis showed a significant increase in the phosphorylation levels of both ERK1 and ERK2 in H9C2 sh-NAGLU compared to control clones (Fig. 5a). Furthermore, while cell treatment with the EGFR inhibitor AG1478 had no effect on ERK1/2 phosphorylation levels in control clones, it promoted a significant reduction of ERK1/2 phosphorylation in H9C2 sh-NAGLU as compared to untreated clones (Fig. 5b), thus suggesting that ERK1/2 activation in NAGLU-depleted cardiomyoblasts is mainly EGFR dependent. Interestingly, H9C2 sh-NAGLU treatment with PD98059^34,35^, a selective inhibitor of the MEK/ERK pathway, caused a significant reduction of ANP and BNP mRNA levels (Fig. 5c, d), indicating that ERK1/2 phosphorylation is essential for the hypertrophic response in NAGLU-depleted H9C2. These results were confirmed by phalloidin staining of both H9C2 sh-CTR and H9C2 sh-NAGLU exposed to PD98059 inhibitor. PD98059 treatment did not affect cell size of H9C2 sh-CTR clones(Fig. 6a, upper panels), whereas it caused a significant reduction of cell dimensions of H9C2 sh-NAGLU clones (Fig. 6a, lower panels). Furthermore, we asked whether MEK/ERK pathway inhibition would also rescue the lysosomal storage phenotype, and we evaluated lysosomal compartments in PD98059-treated H9C2 sh-CTR and H9C2 sh-NAGLU. PD98059 treatment did not affect lysosome staining in H9C2 sh-CTR clones (Fig. 6b, upper panels), whereas it caused a significant reduction of the lysosomal storage defect in H9C2 sh-NAGLU clones (Fig. 6b, lower panels).

Overall, these data indicate that ERK1/2 phosphorylation mediates EGFR-induced hypertrophic and lysosomal phenotypes in NAGLU-depleted H9C2.

### NAGLU depletion in H9C2 cardiomyoblasts promotes c-Src activation

**Fig. 7:**
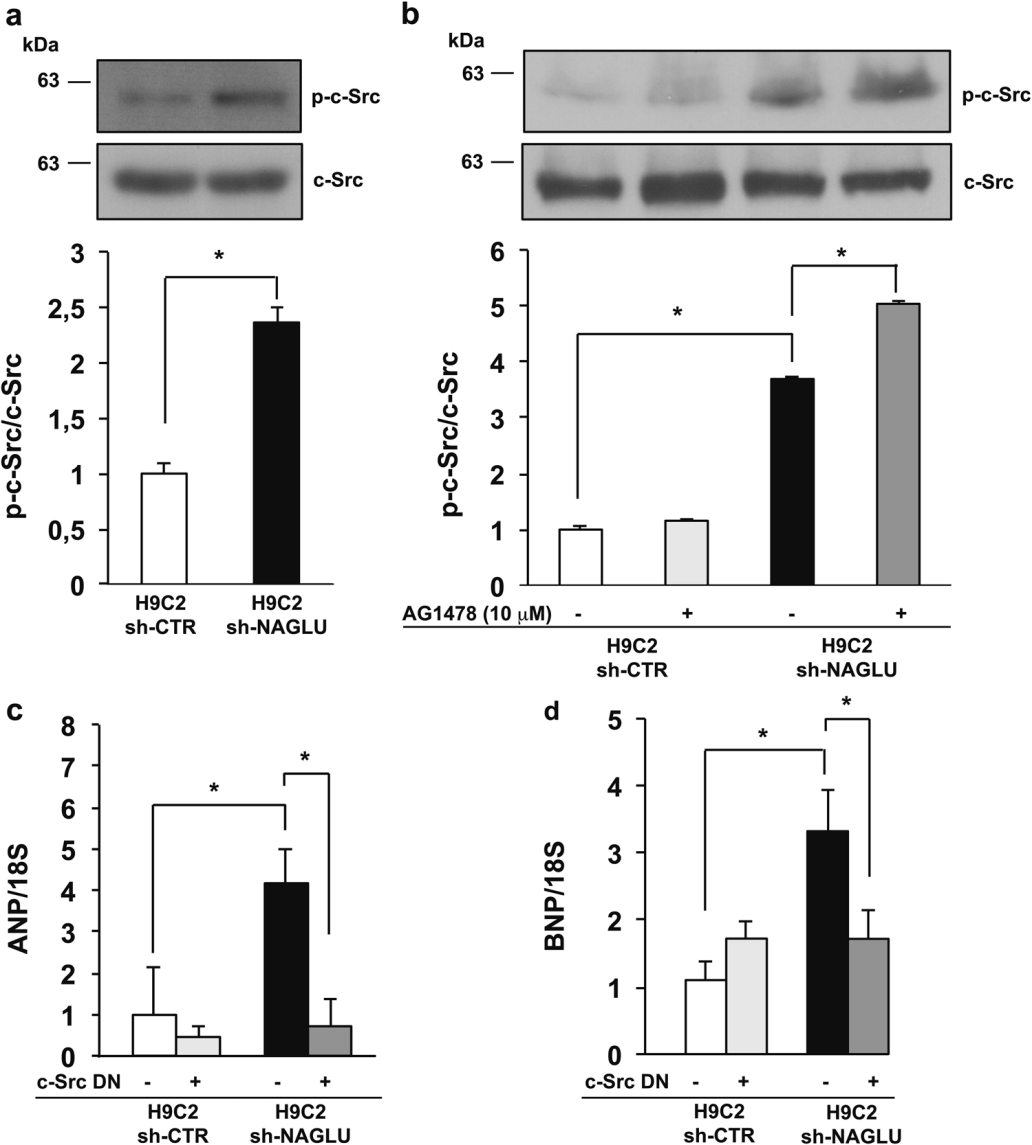
NAGLU silencing induces c-Src activation in H9C2 cardiomyoblasts. **a** c-Src phosphorylation levels in H9C2 sh-CTR and H9C2 sh-NAGLU as measured by western blotting analysis. The upper blot was stripped and reprobed using anti-c-Src antibody. The data reported are the mean ± SD of three independent experiments of equal design. Densitometric analysis of the bands was performed, and the data obtained are reported on the histogram below. *P < 0.05. **b** c-Src phosphorylation levels in H9C2 sh-CTR and H9C2 sh-NAGLU, both untreated and treated for 90 min with 10 μM the EGFR inhibitor AG1478 as measured by western blotting analysis. The upper blot was stripped and reprobed using anti-c-Src antibody. The data reported are the mean ± SD of three independent experiments of equal design. Densitometric analysis of the bands was performed, and the data obtained are reported on the histogram below. *P < 0.05. **c**, **d** Expression levels of mRNAs coding for ANP (**c**) and BNP (**d**) in H9C2 sh-CTR and H9C2 sh-NAGLU, both transfected with DN c-Src as measured by quantitative RT-PCR analysis. The amount of ANP and BNP mRNA was normalized with respect to the amount of 18S ribosomal RNA housekeeping gene. The data reported are the mean ± SD of three independent experiments. *P < 0.05

Multiple evidence supports a direct link between EGFR signaling and enhanced Src-family kinase activity^36–38^. Here, we evaluated the phosphorylation levels of c-Src in both H9C2 sh-NAGLU and control clones. The results obtained showed a significant increase of c-Src phosphorylation in NAGLU-depleted H9C2 as compared to H9C2 sh-CTR (Fig. 7a). However, H9C2 sh-NAGLU treatment with the EGFR inhibitor AG1478 did not reduce c-Src phosphorylation levels (Fig. 7b), suggesting that c-Src phosphorylation represents an upstream event in EGFR activation. Indeed, the transfection of H9C2 sh-CTR and H9C2 sh-NAGLU clones with a dominant-negative (DN) c-Src, while had no effects on H9C2 sh-CTR clones, caused the reduction of EGFR phosphorylation in the H9C2 sh-NAGLU as detected by western blotting analysis (Supplementary Figure S3a), indicating that EGFR phosphorylation in our cellular model of MPS IIIB might be mediated also by c-Src. Furthermore, the involvement of c-Src activation in the hypertrophic phenotype of NAGLU-depleted cardiomyoblasts was evaluated by measuring ANP and BNP mRNA levels in H9C2 sh-NAGLU transfected with DN or (wild-type) WT c-Src. A significant reduction of ANP and BNP mRNA levels was observed in H9C2 sh-NAGLU in the presence of DN c-Src (Fig. 7c, d), whereas ANP and BNP mRNA levels were strongly increased in the presence of WT c-Src (Supplementary Figure S3c, d). By contrast, transfection with DN c-Src or WT c-Src had no effect on either EGFR phosphorylation or ANP and BNP mRNA levels in H9C2 sh-CTR.

These results indicate that c-Src activation contributes to the hypertrophic response in NAGLU-depleted cardiomyoblasts by activating EGFR and its downstream signaling.

### Heparin-binding EGF-like growth factor (HB-EGF) upregulation in NAGLU-depleted H9C2

**Fig. 8:**
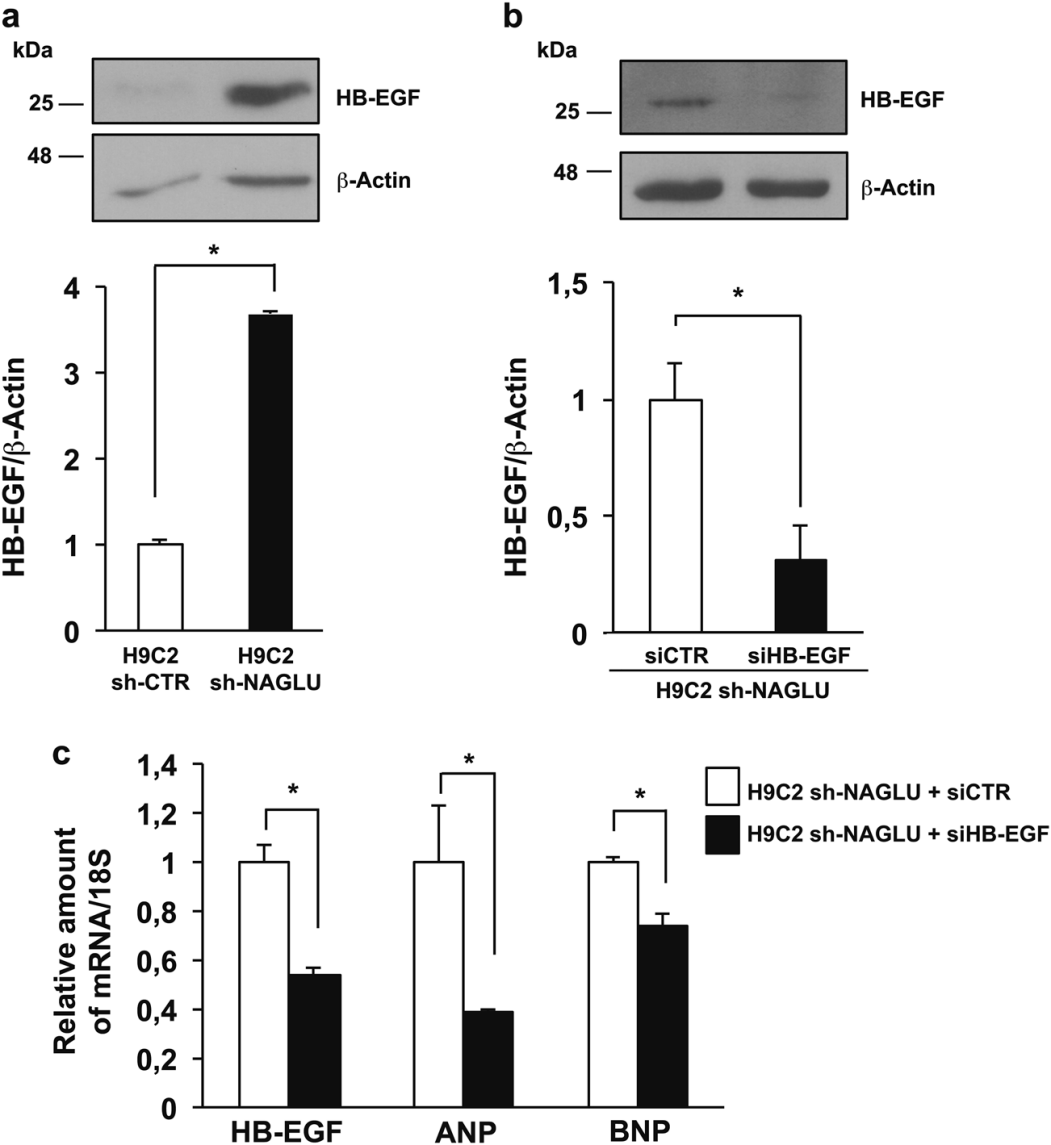
Heparin-binding EGF-like growth factor (HB-EGF) upregulation in NAGLU-silenced H9C2 cardiomyoblasts. **a** HB-EGF protein expression levels in H9C2 sh-CTR and H9C2 sh-NAGLU as measured by western blotting analysis. The amount of HB-EGF as measured by densitometry was normalized with respect to the amount of β-actin. The data reported are the mean ± SD of three independent experiments. *P < 0.05. **b** HB-EGF protein levels in NAGLU sh-H9C2 transfected with HB-EGF siRNA (siHB-EGF) and with a non-targeting siRNA (siCTR) as measured by western blotting analysis. To monitor equal loading of protein in the gel lanes, the blot was probed using anti-β-actin antibody (lower blot). Densitometric analysis of the bands was performed, and the data obtained are reported on the histogram below. The data reported are the mean ± SD of three independent experiments. *P < 0.05. **c** Expression levels of mRNAs coding for HB-EGF, ANP, and BNP in H9C2 sh-NAGLU transfected with HB-EGF siRNA (siHB-EGF) and with a non-targeting siRNA (siCTR) as measured by quantitative RT-PCR analysis. The amount of HB-EGF, ANP, and BNP mRNAs were normalized with respect to the amount of 18S ribosomal RNA housekeeping gene. The data reported are the mean ± SD of three independent experiments. *P < 0.05

It has been well established that the progressive accumulation of HSPGs on the cell membrane and extracellular matrix contributes to the pathogenesis of MPSs^10–14^. The extracellular HSPGs can bind and regulate the activity of HB-EGF that is a potent EGFR ligand, and EGFR activation by HB-EGF plays a role in the hypertrophic signaling in cardiomyocytes^39,40^. We investigated by western blot analysis the expression levels of HB-EGF in our cellular model, and we detected a significant increase of HB-EGF protein expression in H9C2 sh-NAGLU as compared to control clones (Fig. 8a). Furthermore, we tested whether HB-EGF silencing with a specific siRNA would result in the reduction of the EGFR-induced hypertrophic response. Upon HB-EGF silencing in the H9C2 sh-NAGLU (Fig. 8b, c), we detected the reduction of ANP and BNP mRNA levels compared to the same cells treated with a scrambled siRNA (Fig. 8c).

Overall, these findings, together with c-Src-dependent EGFR transactivation, suggest that NAGLU-deficient cardiomyoblasts develop a hypertrophic phenotype through a coordinated intracellular and extracellular EGFR activation induced by c-Src and HB-EGF, respectively.

## Discussion

In this study, we provide the first evidence that abnormal HS accumulation in a MPS IIIB cell model is associated with the activation of EGFR signaling resulting in a hypertrophic phenotype. We first established that NAGLU silencing in H9C2 rat cardiomyoblasts fully recapitulates the MPS IIIB lysosomal phenotype and reproduces the hypertrophic phenotype previously detected in the cardiac tissues of NAGLU knockout mice^24^. These results confirm the reliability of our MPS IIIB cell model to investigate the molecular events induced by NAGLU silencing in cardiac cells.

Cardiac hypertrophy is the common response of the heart to different stimuli, however, prolonged hypertrophy increases the risk of heart failure or sudden cardiac death^41,42^. Increasing evidence is emerging on the involvement of EGFR pathway activation in the development of cardiac hypertrophy^43,44^. Consistently, we found the specific activation of EGFR in NAGLU-depleted cardiomyoblasts. Furthermore, we demonstrated that EGFR inhibition is able to prevent both hypertrophy and lysosomal defects in NAGLU-silenced cardiomyoblasts, suggesting an active role of EGFR in the development of cell pathology in MPS IIIB disease. The rescue of lysosomal phenotype in NAGLU knockdown cardiomyoblasts following EGFR inhibition might be ascribed to the block of EGFR signaling cascade regulating the activity of particular transcription factors involved in GAG biosynthesis^30–32^. Indeed, a therapeutic option for MPSs, called substrate reduction therapy, is based on the use of genistein, a compound belonging to the family of isoflavones, which impairs GAG synthesis by inhibiting EGFR kinase activity. Genistein treatment, however, only partially ameliorates the clinical signs of MPS IIIB disease due to its not exclusive action toward EGFR^32,45^. These findings highlight the need for more studies aimed to elucidate the precise signaling pathways involving EGFR in the cellular events consequent to the abnormal HS accumulation in MPS IIIB, including those effects that lead to cardiac hypertrophy.

The pathogenesis of cardiac hypertrophy involves a number of complex molecular mechanisms including Wnt/Frizzled signals, calcineurin, mitofusin-2, tumor necrosis factor-α, mitogen-activated protein kinases, Rho kinase, Jun kinases (JNK), poly (ADP-ribose) polymerase, transcription factors, oxidative signals, and G protein-coupled-receptor-associated signaling systems^45^. However, the MEK1/ERK1/2 pathway occupies a central regulatory position in the signaling hierarchy that promotes cardiac hypertrophy^33^. On the other hand, the major actors of cardiac hypertrophy, such as ERKs and JNKs, are activated by EGFR^46,47^. In agreement with the above evidence, NAGLU-depleted cardiomyoblasts showed enhanced ERK1/2 phosphorylation, which was inhibited by the EGFR inhibitor AG1478. Interestingly, inhibition of ERK1/2 phosphorylation by the MEK/ERK inhibitor PD98059 strongly reduced ANP and BNP mRNA expression levels as well as lysosomal storage defects in NAGLU-depleted cardiomyoblasts, thus indicating that ERK1/2 phosphorylation induced by EGFR activation is essential for the lysosomal and hypertrophic phenotypes detected in our MPS IIIIB cell model.

Many EGFR-mediated cellular functions require the interaction of the receptor with cellular Src (c-Src), a membrane-associate tyrosine kinase^36,37,48^. The interactions between EGFR and c-Src are bi-directional, in the sense that c-Src can bind, phosphorylate and activate EGFR and vice versa. The consequence of this interaction is an enhanced phosphorylation of specific substrates. For instance, c-Src-dependent transactivation of EGFR and Akt/MAPKs is essential for COX-2 upregulation in endothelial cells^38^. Furthermore, c-Src activation and its association with transactivated EGFR have been also detected in cells stimulated with non-EGF agonists^37^. In the present study, we found increased phosphorylation levels of c-Src in NAGLU-depleted cardiomyoblasts vs. control clones, while c-Src phosphorylation resulted to be unaffected by treatment with the EGFR inhibitor AG1478, suggesting that c-Src phosphorylation represents an upstream event in EGFR activation. Indeed, EGFR phosphorylation levels decreased in H9C2 sh-NAGLU cardiomyoblasts transfected with a DN c-Src. Moreover, ANP and BNP mRNA expression levels resulted to be downregulated or upregulated in H9C2 sh-NAGLU cardiomyoblasts transfected with DN or WT c-Src, respectively. These findings indicate that, in our MPS IIIB cell model, c-Src phosphorylation might mediate EGFR activation with subsequent signaling leading to the hypertrophic phenotype. Given that c-Src is required for EGFR internalization and signaling, and that active c-Src may induce ligand-independent EGFR activation^49^, inhibition of c-Src might also impede EGFR signaling events in NAGLU defective cells as well as in other cells lacking a lysosomal enzyme involved in GAG metabolism.

Commonly, EGFR activation may occur either by transactivation or by binding with ligands such as EGF and HB-EGF. Transactivation of RTKs by G protein-coupled receptors (GPCRs) is a common pathway for transmission of reactive oxygen species-sensitive signals, and interaction between GPCRs and RTKs may occur either in a ligand-independent manner involving membrane-associated non-receptor tyrosine kinases, such as c-Src, or through the activation of metalloproteases (MMPs), which induce the release of RTK ligands such as HB-EGF^50,51^. On the other hand, it is well established that extracellular HSPGs, whose accumulation at the cell membrane and extracellular matrix contributes to the pathogenesis of MPSs, sequester soluble secreted growth factors and facilitate their binding to the cognate receptors, thus activating receptor signaling^15–17^. However, HSPGs also interact with growth factors that remain anchored to the cell surface via a transmembrane domain, and especially those belonging to the EGFR-ligand family which include HB-EGF^39^. The proteolytic cleavage of the anchored form of HB-EGF, namely proHB-EGF, at the extracellular domain gives rise to the soluble form of HB-EGF. Recently, an important role for HB-EGF in cardiac heart development, as well as in cardiac hypertrophy, has been demonstrated^40,52,53^. Indeed, angiotensin II-mediated transactivation of EGFR appears to involve Src-mediated MMP-activated release of HB-EGF^54^. In our investigation, we found the upregulation of HB-EGF in H9C2 sh-NAGLU cardiomyoblasts and the reduction of the hypertrophic response following HB-EGF silencing, suggesting that, in our MPS IIIB cell model, HB-EGF concurs to the activation of EGFR and subsequent hypertrophic signaling. These findings also suggest that inhibition of MMPs that activate EGFR by releasing its ligand HB-EGF might represent a therapeutic strategy for the treatment of lysosomal storage diseases as MPSs. However, further studies are needed to address the role of HB-EGF in MPS IIIB cardiac pathology.

**Fig. 9:**
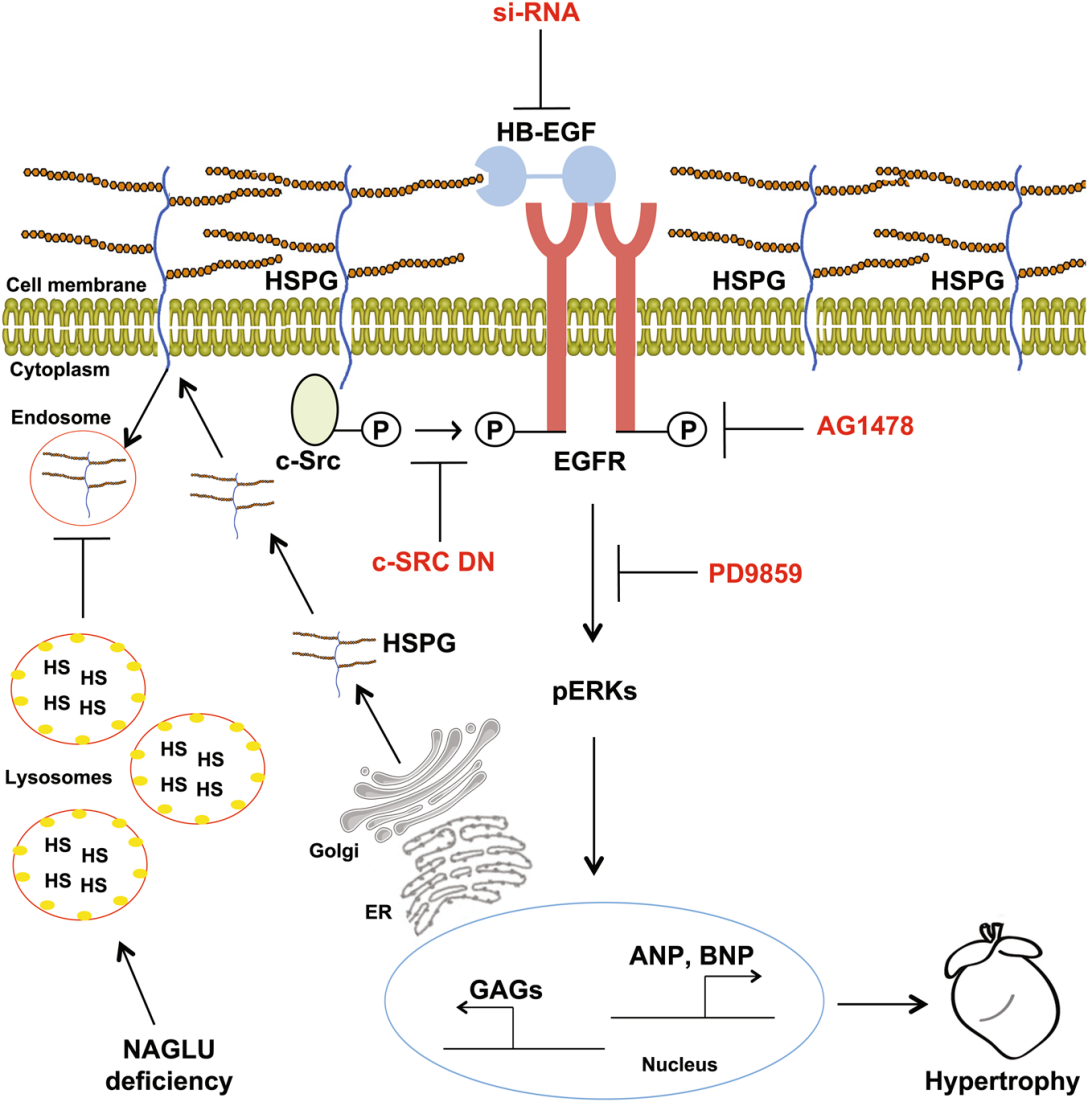
Schematic diagram depicting the signaling pathways by which NAGLU silencing and subsequent HSPG accumulation promote hypertrophy in H9C2 cardiomyoblasts through EGFR

While studies are in progress by the use of the mouse model of the disease to validate in vivo the results obtained in NAGLU-silenced cardiomyoblasts, our findings unravel the cellular signaling pathways by which NAGLU silencing, associated to lysosomal defects and to an excess of extracellular HSPGs, promotes hypertrophy through c-Src- and HB-EGF-mediated activation of EGFR linking to ERK1/2 pathway (Fig. 9). This molecular mechanism that underlies cell pathology consequent to the abnormal HS accumulation in MPS IIIB disease is likely involved in the onset of the cardiac disorders observed in the affected patients. However, we cannot rule out that similar mechanisms may be active in other tissues and organs of MPS IIIB-affected children, and mainly that they may operate in the pathogenesis of other diseases where abnormal GAGs accumulate.

In conclusion, our findings provide novel clues for EGFR pathway targeting-based approaches for MPS IIIB as well as for other lysosomal storage diseases.

## Materials and methods

### Antibodies and chemicals

#### Primary antibodies

Mouse anti-diphosphorylated ERK1/2 monoclonal antibody (M8159, Sigma-Aldrich, St. Louis, MO, USA); rabbit anti-ERK1/2 polyclonal antibody (V114A, Promega, Madison, WI, USA); rabbit anti-HB-EGF monoclonal antibody (ab185555) and rabbit anti-NAGLU monoclonal antibody (ab214671) (Abcam, Cambridge, MA, USA); rabbit anti-phospho-Src (Tyr416) polyclonal antibody (#2101) and rabbit anti-Src (36D10) monoclonal antibody (#2109) (Cell Signaling Technology, Leiden, the Netherlands); mouse anti-β-actin monoclonal antibody (G043, Abm, Richmond, Canada).

#### Secondary antibodies

Goat anti-mouse IgG polyclonal antibody conjugated to horseradish peroxidase (HRP) (sc-2031) and goat anti-rabbit IgG-HRP polyclonal antibody (sc-3837) (Santa Cruz Biotechnology, Heidelberg, Germany).

#### Chemicals

4′,6-diamidino-2-phenylindole (DAPI) (D9542, Sigma-Aldrich); SDS-PAGE reagents (Bio-Rad Laboratories, Hercules, CA, USA); PD98059 [2-(2′-amino-3′-methoxyphenyl)-oxanaphthalen-4-one] (513,000, Calbiochem, La Jolla, CA, USA); AG1478 [*N*-(3-chlorophenyl)−6,7-dimethoxy-4-quinazolinamine], (T4182, Sigma-Aldrich); puromycin, streptomycin and penicillin, and bovine serum albumin (BSA) (Sigma-Aldrich); fetal bovine serum (FBS) (GIBCO, Karlsruhe, Germany); LysoTracker (L7528, Thermo Fisher Scientific, Fremont, CA, USA), phalloidin (P1951, Sigma-Aldrich); lectin from *Triticum vulgaris* (wheat) FITC conjugate (L4895, Sigma-Aldrich); IBAfect reagent (7-2005-050, IBA Lifesciences, Goettingen, Germany); 4-methylumbelliferyl-N-acetyl-α-D-glucosaminide (474,500, Calbiochem); Trizol reagent (15596026, Invitrogen, Carlsbad, CA, USA).

### Cell culture and transfections

H9C2 rat cardiomyoblasts (CRL-1446, ATCC, Wesel, Germany) were cultured in Dulbecco’s minimal essential medium (DMEM), 1 g/l low glucose, 2 mM l-glutamine, 1 mM sodium pyruvate, supplemented with 10% FBS, 100 units/ml penicillin, and 100 μg/ml streptomycin, at 37 °C in a humidified 5% CO_2_ atmosphere.

For stable transfections, H9C2 were plated at a density of 5 × 10^5^ cells/100-mm tissue culture dish in antibiotic-free DMEM containing 10% FBS, and incubated for 24 h at 37 °C with 5% CO_2_. In total, 60–70% confluent cells were transfected using IBAfect reagent, according to the manufacturer’s instructions, with a pool of plasmids codifying for three shRNAs (188A12, 566F3, and 526A3) targeting NAGLU or with a control plasmid codifying for a non-targeting shRNA (Open Biosystems, Lafayette, CO, USA). Forty-eight hours later, transfected H9C2 were selected in the presence of 0.4 μg/ml of puromycin and subjected to enzymatic activity assay to identify the stable NAGLU-silenced clones. Stable clones were grown in the same culture medium of H9C2 supplemented with 0.4 μg/ml of puromycin for the all experimentation.

For transient transfections, H9C2 clones were plated at 2.5 × 10^5^ cells/60-mm tissue culture dish 24 h prior to transfection. Transfections with plasmid vectors codifying for c-Src DN and WT, and control vector-GFP^55^, kindly provided by Prof. A. Feliciello (University of Naples Federico II, Naples, Italy), were carried out using IBAfect reagent according to the manufacturer’s instructions. After 48 h, transfected H9C2 clones were collected for molecular analyses.

Transient transfections of H9C2 clones with HB-EGF siRNA (#AM16708, siRNAID 48748, Thermo Fisher Scientific) and with a non-targeting siRNA (#AM4611, Thermo Fisher Scientific) were carried out using Lipofectamine RNAiMAX (#13778100, Thermo Fisher Scientific) and 10 nM siRNA, and after 48 h, transfected H9C2 clones were collected for molecular analyses.

### Enzyme activity assay

To determine NAGLU enzymatic activity in stable clones, pellets from 5 × 10^5^ cells of each clone were collected, submitted to 10 freeze thaw cycles, and clarified by centrifugation. Protein concentration of samples was determined by the Lowry method. NAGLU enzymatic activity of each clone was measured as described by Marsh and Fensom using 4-methylumbelliferyl-*N*-acetyl-α-d-glucosaminide as fluorogenic substrate^56^. Enzymatic activity was normalized for total protein concentration, and hydrolysis of 1 nmol of substrate per hour per milligram of protein was defined as one catalytic unit.

### Fluorescence microscopy

LysoTracker was used to label lysosomes^57^. Briefly, live cell clones grown on a coverslip were incubated with LysoTracker probe for 1 h at 37 °C, washed with PBS, and fixed with 4% paraformaldehyde (PFA) solution in PBS.

Phalloidin was used to label actin cytoskeleton. After washing with PBS, cells were fixed with 4% PFA solution in PBS, permeabilized with 0.1% Triton X-100, and stained with 50 μg/ml TRITC-conjugated phalloidin for 30 min at room temperature. Subsequently, nuclei were counterstained with DAPI for 15 min.

Lectin was used to label HS. After washing with PBS, cells were fixed with 4% PFA solution in PBS. After washing in PBS, cells were permeabilized with 0.1% Triton X-100 in PBS, and stained with 50 μg/ml fluorescent lectin conjugate, lectin-FITC, in PBS for 1 h at room temperature.

After washing with PBS, the coverslips were mounted with 1:1 PBS/glycerol solution, and observed under a confocal fluorescence microscope. Images were collected using a laser-scanning microscope (LSM 510 META, Carl Zeiss Microimaging, Inc.) equipped with a planapo x63 oil immersion (NA 1.4) objective lens using the same setting conditions (e.g., laser power and detector gain) in control and silenced clones.

Morphometric and image quantitative analyses were performed with LSM software. In particular, the drawing tool was used to measure the relative dimensions and cell area. Fluorescence intensity of random chosen areas of same size was measured^58^.

### Quantitative RT-PCR

Total RNA from H9C2 stable clones was extracted using Trizol reagent following the manufacturer’s instruction. Five hundred nanograms of RNA were reverse transcribed for cDNA synthesis with Iscript RT-PCR system (Bio-rad Laboratories). Reverse transcription of the RNAs was followed by quantitative real-time PCR (Q-PCR) performed with the SYBR Green real-time PCR master mix kit (FS Universal SYBR Green MasterRox/Roche Applied Science) as previously described^59^. The reactions were visualized by SYBR Green analysis (Applied Biosystem Inc, Foster City, CA, USA) on StepOne instrument (Applied Biosystem). Primers used for gene analysis were the following: ANP-Forward: 5′-GGAGCAAATCCCGTATACAGTGCG-3′, Reverse: 5′-GCGGAGGCAT GACCTCATCTTCTAC-3′; BNP-Forward: 5′-CTGGGAAGTCCTAGCCAG TCTC-3′; Reverse: 5′-CCGGAAGGCGCTGTCTTGAGACC-3′; HB-EGF-Forward: 5′-GGACTACTGCATCCAC GGAGAGT-3′; HB-EGF-Reverse: 5′-CCACCACAGCCAAGACGGTAGT-3′; NAGLU-Forward: 5′-GGCCAGGAGGCCATCTGGC-3′; Reverse: 5′-CCCAGGCCAGGAA GGCAGG-3′; S18-Forward: 5′-AAACGGCTACCACATCCAAG-3′; Reverse: 5′-CCTCCAAT GGATCCTCGTTAA-3′. All standards and samples were assayed in triplicate. Thermal cycling was initiated with an initial denaturation at 95 °C for 5 min. After this initial step, 40 cycles of PCR were performed. Each PCR cycle consisted of heating at 95 °C for 30 s for melting, 55 °C for 30 s for annealing, and 72 °C for 30 s for the extension. To calculate the relative expression levels, we used the 2^-ΔΔCT^ method ^60^.

### Western blotting

Cells, grown to sub-confluence in standard medium, were harvested in lysis buffer (50 mM Tris pH 7.5, 150 mM NaCl, 1 mM EDTA, 1 mM EGTA, 10% glycerol, 1% Triton X-100, 1 mM β-glycerophosphate, 1 mM phenylmethylsulfonyl fluoride, protease inhibitor cocktail tablet, 1 mM sodium orthovanadate, and 2.5 mM sodium pyrophosphate)^61^. The lysates were incubated for 30 min on ice, and supernatants were collected and centrifuged for 30 min at 14,000 × *g*. Protein concentration was estimated by Bradford assay, and 25 or 50 μg/lane of total proteins were separated on SDS gels and transferred to nitrocellulose membranes^62^. Membranes were treated with a blocking buffer (25 mM Tris, pH 7.4, 200 mM NaCl, 0.5% Triton X-100) containing 5% non-fat powdered milk for 1 h at room temperature^63^. Incubation with the primary antibody was carried out overnight at 4 °C. After washings, membranes were incubated with the HRP-conjugated secondary antibody for 1 h at room temperature. Following further washings of the membranes, chemiluminescence was generated by enhanced chemiluminescence (ECL) kit (Amesharm, Little Chalfont, UK). Densitometric analyses were performed using the NIH Image software (Bethesda, MD, USA).

### Phospho-receptor tyrosine kinase (phospho-RTK) array

The Phospho-RTK Array (ARY001B, R&D Systems, Minneapolis, MN, USA) is a rapid and sensitive tool to detect changes in phosphorylation between samples. The array, which allows the screening of 49 different phosphorylated RTKs, was performed as here briefly described. All reagents and samples were prepared according to the manufacturer’s instructions. An aliquot of 2 ml of Array Buffer 1 was pipetted into each well of the 4-well multi-dish as a blocking buffer; each array was placed into each well of the 4-well multi-dish and incubated for 1 h at room temperature on a rocking platform shaker. Samples were prepared by diluting the desired quantity (50 μg of proteins) of cell lysates to a final volume of 1.5 ml with Array Buffer 1, and incubated overnight at 2–8 °C on a rocking platform shaker pre-blocked membrane. Each array was carefully placed into individual plastic containers with 2 ml of 1× Wash Buffer, and the 4-well multi-dish was rinsed with deionized or distilled water, and dried thoroughly. Each array was washed with 1× Wash Buffer for 10 min on a rocking platform shaker for three times. The anti-phospho-tyrosine-HRP detection antibody was finally diluted in 1× Array Buffer 2, and added to each array for 2 h at room temperature on a rocking platform shaker. Each array was washed three times with 2 ml of 1× Wash Buffer for 10 min on a rocking platform shaker. Membranes were incubated with 1 ml of the prepared Chemi Reagent Mix for 1 min. Membranes were exposed with an autoradiography film cassette to an X-ray film for 1–10 min. For the data analysis, the positive signal seen on developed film was identified by placing the transparency overlay template on the array image and aligning it with the pairs of reference spots in the three corners of each array. Reference spots are included to align the transparency overlay template and to demonstrate that the array has been incubated with anti-phospho-tyrosine-HRP during the assay procedure.

### Statistical analysis

Data reported are expressed as the mean ± SD of at least three separate experiments. Statistical significance was determined by Student’s *t* test. A value of *P* < 0.05 was considered to be statistically significant.

## Electronic supplementary material

Supplementary information

Supplementary Table 1

Supplementary Figure S1

Supplementary Figure S2

Supplementary Figure S3
