# Supplementary material for: EGFR activation triggers cellular hypertrophy and lysosomal disease in NAGLU-depleted cardiomyoblasts, mimicking the hallmarks of mucopolysaccharidosis IIIB
Source: Cell Death Dis. 2018 Jan 18;9(2):40. doi: 10.1038/s41419-017-0187-0 (PMC5833457; doi:10.1038/s41419-017-0187-0)
Supplement: Supplementary file 1 — Supplementary information [file 41419_2017_187_MOESM1_ESM.docx]

**Supplementary Information**

**Title:** EGFR activation triggers cellular hypertrophy and lysosomal disease in NAGLU depleted cardiomyoblasts, mimicking the hallmarks of mucopolysaccharidosis IIIB

**Authors: Authors:** Valeria De Pasquale^1,*^, Antonio Pezone^1,*^, Patrizia Sarogni^1^, Alfonso Tramontano^1^, Gabriele Giacomo Schiattarella^2^, Vittorio Enrico Avvedimento^1^, Simona Paladino^1^, and Luigi Michele Pavone^1^

**Affiliations:** ^1^Department of Molecular Medicine and Medical Biotechnology and ^2^Department of Advanced Biomedical Sciences, University of Naples Federico II, Naples, Italy

**Materials and Methods**

**Antibodies and chemicals.** *Primary antibodies:* rabbit anti-LAMP-2 polyclonal antibody (PA1-655, Thermo-Fisher Scientific, Fremont, CA, USA) rabbit anti-phospho-EGFR (Tyr1173) monoclonal antibody (#4407), and rabbit anti-EGFR (C74B9) monoclonal antibody (#2646) (Cell Signaling Technology, Leiden, The Netherlands). *Secondary antibodies:* goat anti-rabbit IgG-HRP polyclonal antibody (sc-3837, Santa Cruz Biotechnology, Heidelberg, Germany). *Chemicals:* puromicyn, streptomycin, penicillin, and bovine serum albumin (BSA) (A7906) (Sigma Aldrich, St. Louis, MO, USA);, fetal bovine serum (FBS) (GIBCO, Karlsruhe, Germany); IBAfect reagent (7-2005-050, IBA Lifesciences, Goettingen, Germany); Trizol reagent (15596026, Invitrogen, Carlsbad, CA,USA).

**Cell culture and transfections.** H9C2 rat cardiomyoblasts (CRL-1446, ATCC, Wesel, Germany) were cultured in Dulbecco’s minimal essential medium (DMEM), 1 g/l low glucose, 2 mM L-glutamine, 1 mM sodium pyruvate, supplemented with 10 % FBS, 100 Units/ml penicillin, and 10 mg/ml streptomycin, at 37 °C in a humidified 5 % CO_2_ atmosphere. For stable transfection, H9C2 were plated at a density of 5 × 10^5^ cells/100-mm tissue culture dish in antibiotic-free DMEM containing 10 % FBS, and incubated for 24 h at 37 °C with 5 % CO_2_. 60-70 % confluent cells were transfected using IBAfect reagent according to the manufacturer's instructions with a pool of plasmids codifying for three shRNAs targeting NAGLU (188A12, 566F3, 526A3) or with a control plasmid codifying for a non-targeting shRNA (Open Biosystems, Lafayette, CO, USA). 48 h later, transfected H9C2 were selected in the presence of 0.4 μg/ml of puromycin, and subjected to enzymatic activity assay and RT-PCR analysis to identify the stable NAGLU-silenced clones. Stable clones were grown in the same culture medium of H9C2 supplemented with 0.4 μg/ml of puromycin for the all experimentation.

**Coomassie staining.** Cells of each clone were washed in PBS, fixed with 4 % PFA solution in PBS for 1 h at room temperature, then washed in PBS and stained with coomassie brilliant blue solution for 1 h at room temperature. Finally, the cells were washed in distilled water, and dried up for 24 h. Cells were observed under a white light microscope, and photographed with Nikon camera.

**Cell cycle analysis.** H9C2 sh-NAGLU and control clones (H9C2 sh-CTR) were detached by trypsinization, washed in PBS, and fixed in 70 % ethanol. Before analysis, cells were washed in PBS, re-suspended in a PBS solution containing RNase (Roche Life Science, Monza, Italy) and propidium iodide (Sigma Aldrich), and stored in the dark for 20 min at room temperature. Fluorescence was detected using the 488 nm laser line with a CyAn ADP Flow Cytometer (DAKO Cytomation, Milan, Italy). Not less than 12 000 events were recorded for each sample. Cell cycle profiles were analyzed using ModFit/LT 3.2 version (Verity Software, Topsham, ME, USA). Data were obtained from three independent experiments performed in triplicate, and expressed as mean ± S.D.

**Proliferation and differentiation assays.** For proliferation assay, 5×10^5^ cells of each clone were plated in a 60-mm tissue culture dish, and after 24, 36 and 48 h of incubation at 37 °C cells were trypsinized. Cells were labeled with a solution of trypan blue, the number of alive cells was determined by direct counting with a hemocytometer. The data reported are the means of three independent experiments performed with each sample in replicates of three. For differentiation assay, confluent H9C2 clones were grown for one week in 1% FBS, and then processed for ventricular myosin light chain 2 (MLC2V) mRNA level analysis by quantitative RT-PCR.

**Quantitative RT-PCR.** Total RNA from H9C2 stable clones was extracted using Trizol reagent following the manufacturer’s instruction. 500 ng of RNA were reverse transcribed for cDNA synthesis with Iscript RT-PCR system (Bio-rad Laboratories, Hercules, CA, USA). Reverse transcription of the RNAs was followed by quantitative real-time PCR (Q-PCR) performed with the SYBR Green real time PCR master mix kit (FS Universal SYBR Green MasterRox/Roche Applied Science, Pleasanton, CA, USA). The reactions were visualized by SYBR Green analysis (Applied Biosystem Inc., Foster City, CA, USA) on StepOne instrument (Applied Biosystem). Primers for gene analysis were the following: MLC2V-Forward: 5'-GACCCAGATCCAGGAGTTCAAGG-3'; Reverse: 5'-CGAGGGCAGCAAACGTGTCCC-3'; S18-Forward: 5'-AAACGGCTACCAC ATCCAAG-3'; Reverse: 5'-CCTCCAATGGATC CTCGTTAA-3'. All standards and samples were assayed in triplicate. Thermal cycling was initiated with an initial denaturation at 95 °C for 5 min. After this initial step, 40 cycles of PCR were performed. Each PCR cycle consisted of heating at 95 °C for 30 sec for melting, 55 °C for 30 sec for annealing and 72 °C for 30 sec for the extension. To calculate the relative expression levels, we used the 2^-ΔΔCT^ method.

**Western Blotting.** Cells, grown to sub-confluence in standard medium, were harvested in lysis buffer (50 mM Tris ph7.5, 150 mM NaCl, 1 mM EDTA, 1 mM EGTA, 10 % glycerol, 1 % Triton-X-100, 1 mM β-glycerophosphate, 1 mM phenylmethylsulfonyl fluoride, protease inhibitor cocktail tablet, 1 mM sodium orthovanadate, 2.5 mM sodium pyrophosphate). The lysates were incubated for 30 min on ice, and supernatants were collected and centrifuged for 10 min at 14 000 g. Protein concentration was estimated by Bradford assay, and 25 or 50 μg/lane of total proteins were separated on SDS gels and transferred to nitrocellulose membranes. Membranes were treated with a blocking buffer (25 mM Tris, pH 7.4, 200 mM NaCl, 0.5 % Triton X-100) containing 5 % non-fat powdered milk for 1 h at room temperature. Incubation with the primary antibody was carried out overnight at 4 °C. After serial washings, membranes were incubated with the horseradish peroxidase-conjugated secondary antibody for 1 h at room temperature. Following further washings of the membranes, chemiluminescence was generated by ECL system. Densitometric analyses were performed using the NIH Image software (Bethesda, MD, USA).

**Statistical analysis.** Data reported are expressed as the mean ± standard deviation (S.D.) of at least three separate experiments. Statistical significance was determined by Student’s t-test. A value of P < 0.05 was considered to be statistically significant.

**SUPPLEMENTARY FIGURE LEGENDS**

**Figure S1** Cell pathology in NAGLU silenced H9C2 cardiomyoblasts. **(a)** LAMP2 protein expression levels in H9C2 sh-CTR and H9C2 sh-NAGLU as measured by Western blotting analysis. The amount of LAMP2 as measured by densitometry was normalized with respect to the amount of β-actin. The data reported are the mean ± S.D. of 3 independent experiments. *P<0.05. **(b)** Coomassie staining of control and NAGLU-silenced clones. H9C2 sh-NAGLU resulted larger in comparison to control clones. **(c)** Cell cycle profiles of control and NAGLU silenced H9C2 as evaluated by cytofluorimetric assay. Representative cell cycle profiles are shown. Numerical data of the phase cycle reported under the various panels are the average of triplicate measurements (mean ± S.D.). Statistical significance (relative to control): ^*^P <0.05. **(d)** NAGLU silencing decreases cell proliferation. After 24, 36 and 48 h of incubation at 37 °C, the cells were trypsinized, stained with trypan blue, and the number of alive cells, was determined by direct counting with hemocytometer. The data reported are the means of three independent experiments performed with each sample in replicates of three. Error bars indicate standard errors of the means. ^*^P <0.05. **(e)** NAGLU silencing reduces cellular differentiation as measured by MLC2V mRNA levels. MLC2V mRNA expression levels in undifferentiated and differentiated H9C2 sh-CTR and H9C2 sh-NAGLU were measured by quantitative RT-PCR analysis. The amount of MLC2V mRNA was normalized with respect to the amount of 18S ribosomal RNA housekeeping gene. The data reported are the mean ± S.D. of 3 independent experiments. *P<0.05.

**Figure S2** **(a)** Increased levels of EGFR phosphorylation levels in NAGLU-depleted H9C2. EGFR phosphorylation levels in H9C2 sh-CTR and H9C2 sh-NAGLU clones were measured by Western blotting. To monitor protein loading of gel lanes, the upper blot was stripped and re-probed using anti-EGFR antibody. The data reported are the mean ± S.D. of 3 independent experiments of equal design. Densitometric analysis of the bands was performed and the data obtained are reported on the histogram below. *P<0.05. **(b)** LAMP2 protein expression levels after cell treatment with the EGFR inhibitor AG1478. LAMP2 protein expression levels in H9C2 sh-CTR and H9C2 sh-NAGLU, both untreated and treated for 24 h with 10 μM of AG1478 as measured by Western blotting analysis. The amount of LAMP2 as measured by densitometry was normalized with respect to the amount of β-actin. The data reported are the mean ± S.D. of 3 independent experiments. *P<0.05.

**Figure S3** **(a)** Reduction of EGFR phosphorylation levels in NAGLU-depleted H9C2 after transfection with c-Src DN. EGFR phosphorylation levels in H9C2 sh-CTR and H9C2 sh-NAGLU, both transfected with c-Src DN as measured by Western blotting analysis. The amount of EGFR phosphorylation as measured by densitometry was normalized with respect to the amount of total EGFR. The data reported are the mean ± S.D. of 3 independent experiments. *P<0.05. **(b)** Expression levels of mRNAs coding for ANP and BNP in H9C2 sh-CTR and H9C2 sh-NAGLU transfected with wild-type (WT) c-Src, as measured by quantitative RT-PCR analysis. The amount of ANP and BNP mRNA were normalized with respect to the amount of 18S ribosomal RNA housekeeping gene. The data reported are the mean ± S.D. of 3 independent experiments. *P<0.05.
