# Supplementary material for: EGFR activation triggers cellular hypertrophy and lysosomal disease in NAGLU-depleted cardiomyoblasts, mimicking the hallmarks of mucopolysaccharidosis IIIB
Source: Cell Death Dis. 2018 Jan 18;9(2):40. doi: 10.1038/s41419-017-0187-0 (PMC5833457; doi:10.1038/s41419-017-0187-0)
Supplement: Supplementary file 2 — Supplementary Table 1 [file 41419_2017_187_MOESM2_ESM.docx]

**Supplementary Table S1:** Phospho-RTKs coordinates

**Coordinate Receptor Family RTK/Control Coodinate Receptor Family RTK/Control**

A1, A2 Reference Spots ___ D1, D2 Tie Tie-2

A23, A24 Reference Spots ___ D3, D4 NGF R TrkA

B1, B2 EGF R EGF R D5, D6 NGF R TrkB

B3, B4 EGF R ErbB2 D7, D8 NGF R TrkC

B5, B6 EGF R ErbB3 D9, D10 VEGF R VEGF R1

B7, B8 EGF R ErbB4 D11, D12 VEGF R VEGF R2

B9, B10 FGF R FGF R1 D13, D14 VEGF R VEGF R3

B11, B12 FGF R FGF R2α D15, D16 MuSK MuSK

B13, B14 FGF R FGF R3 D17, D18 Eph R EphA1

B15, B16 FGF R FGF R4 D19, D20 Eph R EphA2

B17, B18 Insulin R Insulin R D21, D22 Eph R EphA3

B19, B20 Insulin R IGF-I R D23, D24 Eph R EphA4

B21, B22 Axl Axl E1, E2 Eph R EphA6

B23, B24 Axl Dtk E3, E4 Eph R EphA7

C1, C2 Axl Mer E5, E6 Eph R EphB1

C3, C4 HGF R HGF R E7, E8 Eph R EphB2

C5, C6 HGF R MSP R E9, E10 Eph R EphB4

C7, C8 PDGF R PDGF Rα E11, E12 Eph R EphB6

C9, C10 PDGF R PDGF Rβ E13, E14 Insulin R ALK

C11, C12 PDGF R SCF R E15, E16 ___ DDR1

C13, C14 PDGF R Flt-3 E17, E18 ___ DDR2

C15, C16 PDGF R M-CSF R E19, E20 Eph R EphA5

C17, C18 RET c-Ret E21, E22 Eph R EphA10

C19, C20 ROR ROR1 F1, F2 Reference Spots ___

C21, C22 ROR ROR2 F5, F6 Eph R EphB3

C23, C24 Tie Tie-1 F7, F8 ___ RYK

F23, F24 Control(-) PBS
