# Supplementary figures and images for: EGFR activation triggers cellular hypertrophy and lysosomal disease in NAGLU-depleted cardiomyoblasts, mimicking the hallmarks of mucopolysaccharidosis IIIB
Source: Cell Death Dis. 2018 Jan 18;9(2):40. doi: 10.1038/s41419-017-0187-0 (PMC5833457; doi:10.1038/s41419-017-0187-0)

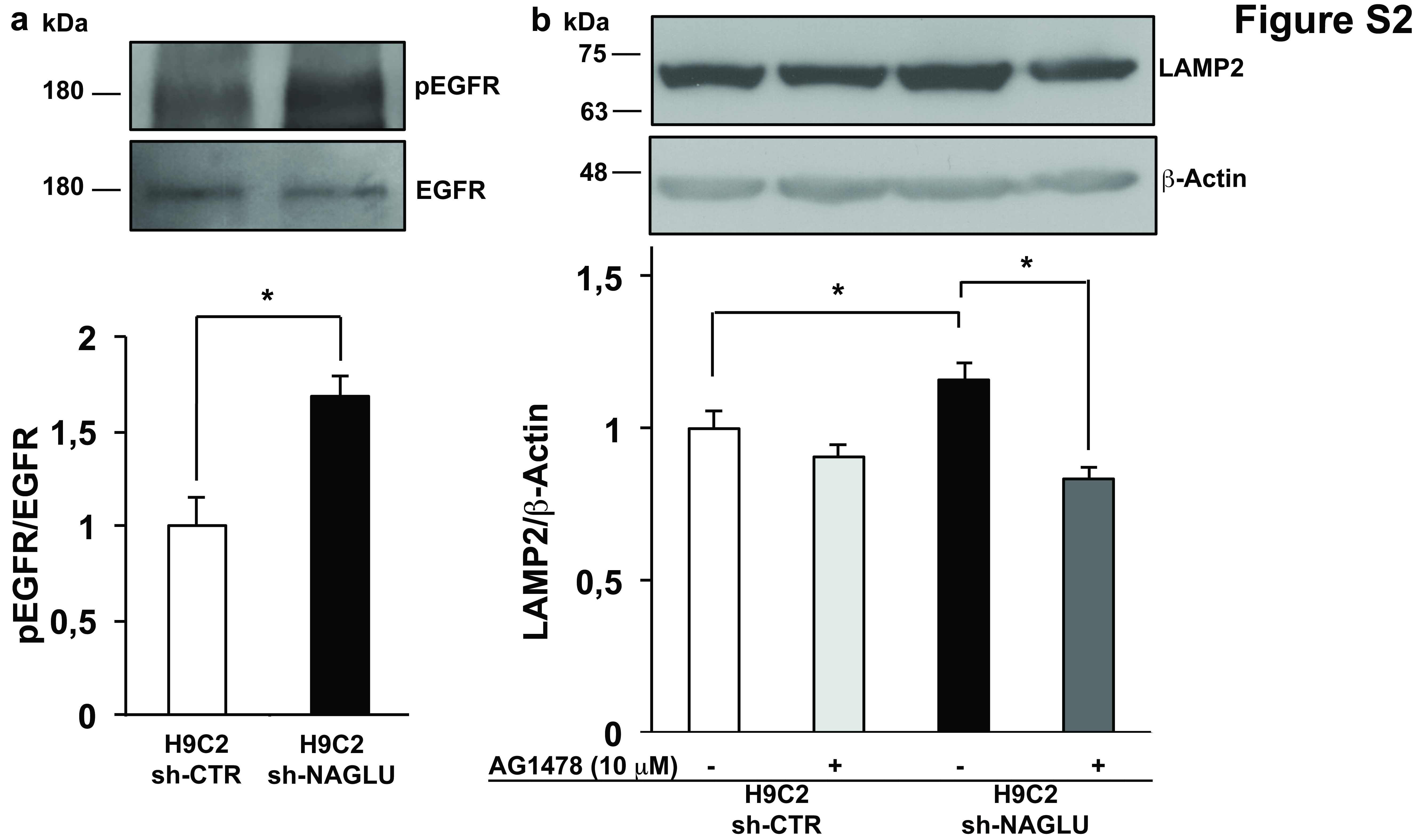

Supplement: Supplementary file 4 — Supplementary Figure S2 [file 41419_2017_187_MOESM4_ESM.tif]
